# A Leak Beneath the Graft: Description of a Rare Ureteral Injury Following Aortobifemoral Bypass

**DOI:** 10.7759/cureus.101773

**Published:** 2026-01-18

**Authors:** Sammy I Khalouf, Jedediah Bondy, Byron Chen, Hakki Celik, Ryan Sutherland

**Affiliations:** 1 Urology, Lake Erie College of Osteopathic Medicine, Erie, USA; 2 Radiology, Lake Erie College of Osteopathic Medicine, Erie, USA; 3 Radiology, UMass Memorial Health, Worcester, USA

**Keywords:** aortobifemoral bypass, ct urography, delayed-phase imaging, multidisciplinary management, retroperitoneal fluid collection, ureteral injury, urinoma, vascular graft complications

## Abstract

Ureteral injury is an uncommon but clinically significant complication of major vascular surgery, including aortobifemoral bypass, due to the close anatomic relationship between the ureters and the aortoiliac vessels. Delayed diagnosis may lead to urinoma formation, urinary obstruction, infection, and contamination of prosthetic vascular grafts; however, clinical presentation is often nonspecific and may resemble more common postoperative vascular complications.

We report a 59-year-old man who underwent elective aortobifemoral bypass with Dacron graft placement and presented two weeks later with progressive left lower quadrant pain radiating to the flank and testicle. Contrast-enhanced computed tomography angiography demonstrated a rim-enhancing retroperitoneal fluid collection encasing the vascular graft with mild hydronephrosis, and delayed-phase CT urography confirmed contrast extravasation from the left ureter, consistent with urinoma due to ureteral injury. Management consisted of multidisciplinary consultation, urinary diversion via percutaneous nephrostomy, avoidance of direct drainage due to graft proximity, and intravenous antibiotic therapy. The patient remained clinically stable with preserved renal function, imaging demonstrated resolution of the urinoma, and a ureteral stent was placed to facilitate healing. This case highlights the importance of considering ureteral injury after vascular bypass surgery and underscores the diagnostic value of delayed-phase CT urography, as early recognition and coordinated multidisciplinary management are essential to prevent renal and graft-related complications.

## Introduction

Ureteral injury is an uncommon yet potentially serious complication of major vascular surgery, including aortobifemoral bypass and abdominal aortic reconstruction. Because the ureters course in close proximity to the aorta and iliac vessels, they are vulnerable to direct trauma, ischemic injury, or delayed compromise from postoperative inflammation or fibrosis during vascular interventions [1,2]. Risk factors that further increase susceptibility include extensive retroperitoneal dissection, distorted anatomy from prior surgery or inflammation, severe atherosclerotic disease, prolonged operative time, and the presence of prosthetic vascular grafts [2]. Although the reported incidence of ureteral injury in vascular surgery is low (6%), failure to recognize this complication can result in significant morbidity, including urinoma formation, urinary obstruction, infection, retroperitoneal fibrosis, and secondary contamination of prosthetic vascular grafts [3].

Clinical presentation is often delayed and nonspecific, with patients reporting abdominal, flank, groin, or testicular pain that may overlap with more common postoperative vascular complications such as graft thrombosis, pseudoaneurysm, anastomotic leak, hematoma, or abscess [4,5]. As a result, ureteral injury may not be immediately considered in the differential diagnosis. Initial imaging in this setting frequently consists of contrast-enhanced computed tomography angiography to assess graft patency and exclude vascular causes; however, standard arterial-phase imaging may provide limited evaluation of the urinary tract and fail to identify urinary leaks [6].

Delayed-phase computed tomography urography plays a critical role in the detection of ureteral injury by allowing visualization of contrast excretion through the collecting system and ureters, thereby enabling identification of contrast extravasation into adjacent fluid collections [6]. Accurate differentiation of a urinoma from other postoperative retroperitoneal collections is essential, particularly when the collection lies adjacent to a prosthetic graft, as management strategies differ significantly and inappropriate intervention may increase the risk of graft infection [3,4].

We present a case of delayed ureteral injury with urinoma formation following aortobifemoral bypass, highlighting the diagnostic value of delayed-phase CT urography and the importance of early recognition and coordinated multidisciplinary management to preserve renal function and prevent graft-related complications.

## Case presentation

A 59-year-old man with a medical history significant for coronary artery disease, hypertension, hyperlipidemia, emphysema, and chronic alcohol use disorder underwent elective aortobifemoral bypass with Dacron graft placement and left femoral endarterectomy for peripheral arterial disease. The immediate postoperative course was uncomplicated, and the patient was discharged in stable condition.

Two weeks after surgery, the patient presented with progressively worsening left lower quadrant abdominal pain radiating to the ipsilateral flank and testicle. He denied fever, chills, dysuria, hematuria, nausea, vomiting, or changes in bowel habits. On presentation, he was afebrile and hemodynamically stable. Physical examination was notable for localized abdominal tenderness without peritoneal signs. Laboratory evaluation revealed a normal serum creatinine level and unremarkable urinalysis.

Although no intraoperative ureteral injury was reported, the delayed onset of symptoms, the location of the leak adjacent to the vascular graft, and the absence of immediate postoperative urinary abnormalities suggest that the injury was more likely related to ischemic or traction-related compromise or delayed extrinsic compression from postoperative inflammation or fibrosis rather than an acute transection. Among these possibilities, an ischemic or traction-related mechanism is favored, given the delayed presentation and the imaging-confirmed mid-to-distal ureteral leak, which is consistent with delayed ureteral necrosis rather than an immediately apparent transection. No intraoperative documentation or histopathologic confirmation was available; therefore, the proposed mechanism is inferred from the clinical timeline and radiographic findings.

**Figure 1:**
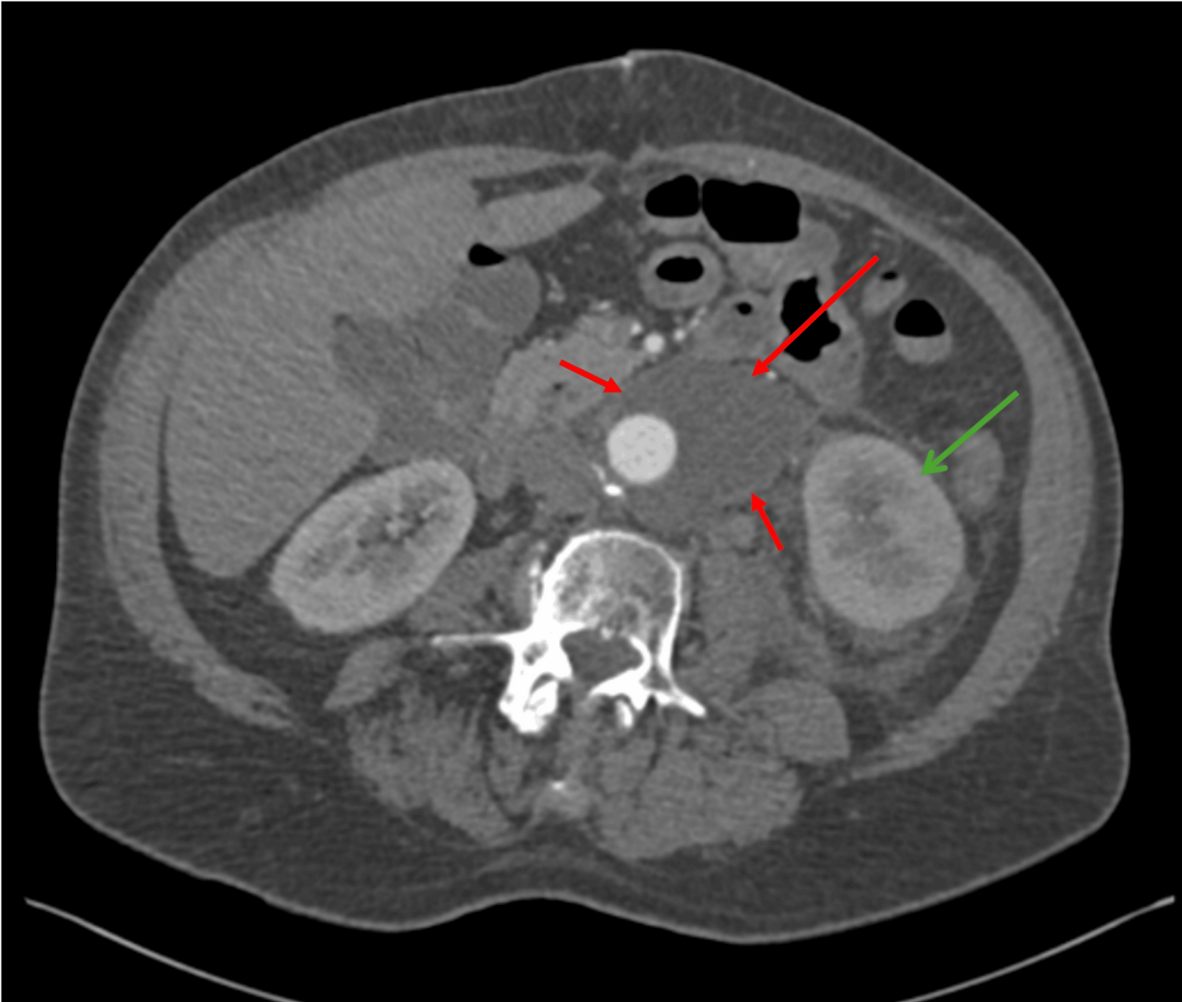
Axial CTA of the abdomen and pelvis with IV contrast (arterial phase) demonstrates retroperitoneal rim-enhancing collection encasing the aortic graft (red arrows) with mild left hydronephrosis (green arrow).

Given concern for postoperative vascular complications, contrast-enhanced computed tomography angiography (CTA) of the abdomen and pelvis was obtained. Imaging demonstrated a multiloculated, rim-enhancing retroperitoneal fluid collection encasing the aortic graft, associated with mild left-sided hydronephrosis and delayed enhancement of the left kidney (Figure 1). The left ureter was poorly visualized along its course through the collection, raising concern for possible ureteral involvement.

**Figure 2:**
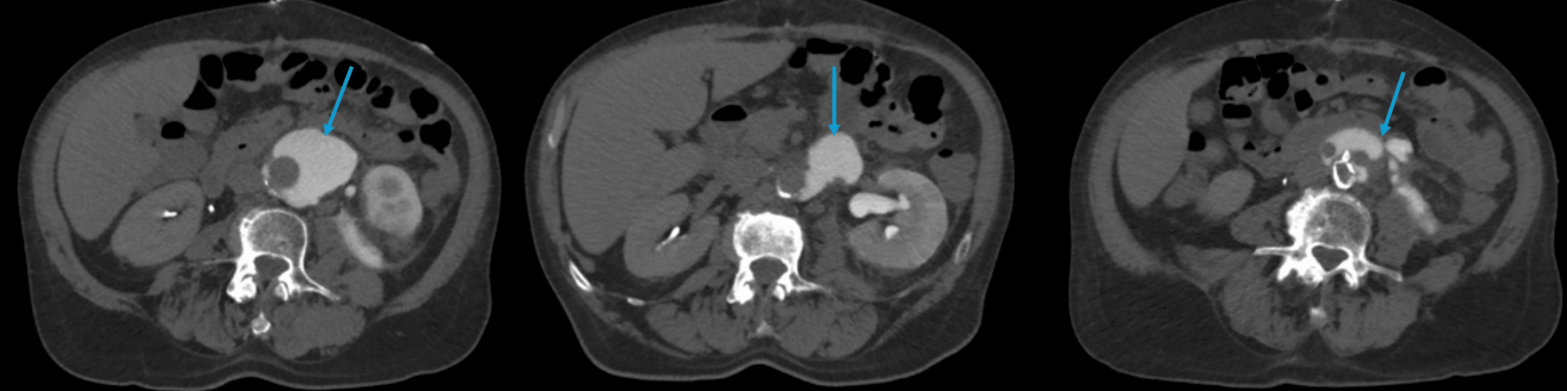
Axial CT urogram (delayed phase) showing contrast extravasation from the left ureter into the retroperitoneal collection (blue arrows), consistent with urinoma formation adjacent to the vascular graft.

To further evaluate the urinary tract, delayed-phase CT urography was performed. This study revealed contrast extravasation from the left ureter into the retroperitoneal fluid collection, confirming a diagnosis of urinoma secondary to ureteral injury (Figure 2).

**Figure 3:**
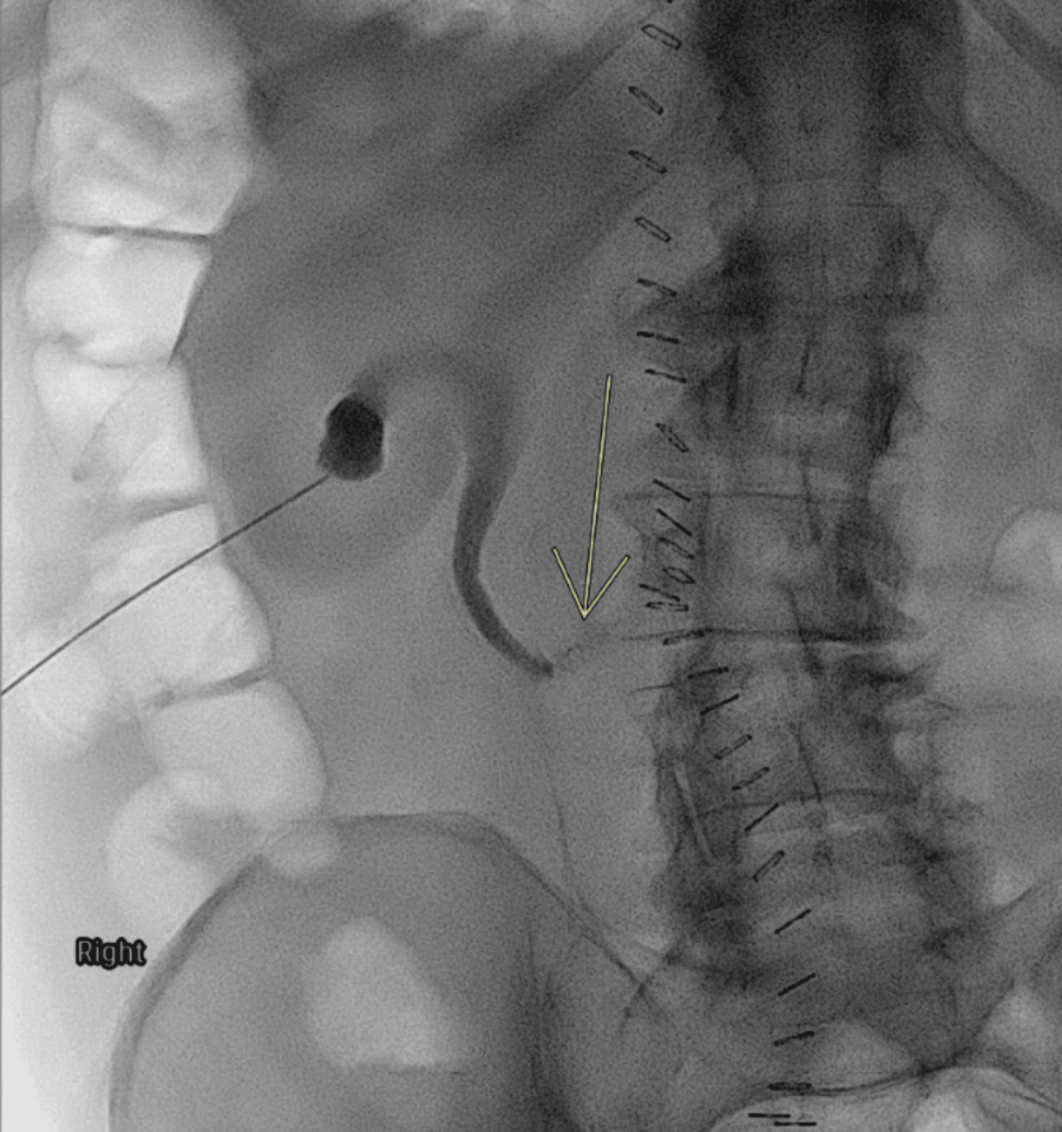
Fluoroscopic nephrostogram via left percutaneous nephrostomy demonstrating contrast extravasation from the mid-to-distal ureter into the retroperitoneum (yellow arrow), confirming ureteral injury.

Multidisciplinary consultations were obtained with urology, interventional radiology, vascular surgery, and infectious disease. Urinary diversion was achieved through placement of a left percutaneous nephrostomy tube to reduce ongoing urine leakage and facilitate ureteral healing (Figure 3). Due to the close proximity of the urinoma to the prosthetic vascular graft, direct percutaneous drainage of the collection was deferred to minimize the risk of graft contamination. Broad-spectrum intravenous antibiotics were initiated for graft protection as recommended by the infectious disease service.

Throughout hospitalization, the patient remained afebrile with preserved renal function and negative blood and urine cultures. Serial imaging over the following weeks demonstrated near-complete resolution of the urinoma. Once the collection had sufficiently resolved, a left ureteral stent was placed to ensure continued urinary drainage and promote definitive ureteral healing. The patient completed a six-week course of intravenous antibiotics and was discharged in stable condition with close outpatient urology follow-up. At follow-up, imaging confirmed complete resolution of the urinoma, and the nephrostomy tube remained patent and functional.

## Discussion

Ureteral injury following major vascular surgery is an uncommon but clinically important complication, with an incidence reported to be less than 6% [3]. The left ureter is more frequently affected due to its close anatomic relationship with the aorta and left common iliac artery [7]. Mechanisms of injury include direct transection or ligation, ischemic compromise from devascularization, excessive traction during dissection, and delayed injury from postoperative inflammation or fibrosis [7]. Although rare, unrecognized ureteral injury can lead to significant morbidity, including urinoma formation, infection, progressive urinary obstruction, and contamination of adjacent prosthetic vascular grafts [3].

Postoperative abdominal, flank, or groin pain following aortobifemoral bypass is more commonly attributed to vascular complications such as graft thrombosis, pseudoaneurysm, anastomotic leak, hematoma, or abscess [4,5]. As a result, ureteral injury may not be initially considered in the differential diagnosis, particularly when laboratory findings and vital signs are unremarkable. In this setting, contrast-enhanced computed tomography angiography (CTA) is frequently the first-line imaging modality because it allows rapid assessment of graft patency and vascular integrity [8]. However, standard arterial-phase imaging provides limited evaluation of the urinary tract and may fail to identify urinary leaks, especially when contrast extravasation is subtle or delayed.

Delayed-phase CT urography plays a critical diagnostic role in the evaluation of suspected ureteral injury by allowing visualization of contrast excretion through the renal collecting system and ureters [8,9]. This imaging phase is typically acquired several minutes after contrast administration and is essential for detecting contrast extravasation into adjacent fluid collections. Without delayed-phase imaging, urinomas may be misinterpreted as abscesses or hematomas, particularly when rim enhancement is present [6]. In the present case, delayed-phase CT urography was pivotal in distinguishing a urinoma from other postoperative retroperitoneal collections and establishing the correct diagnosis.

Several evidence-based strategies have been proposed to reduce the risk of ureteral injury during aortoiliac and aortobifemoral procedures. Preoperative cross-sectional imaging, including CT urography, can help delineate ureteral anatomy in patients with prior surgery, distorted retroperitoneal planes, or severe atherosclerotic disease, thereby allowing surgeons to anticipate areas of vulnerability. Intraoperative ureteral stent placement has also been shown to facilitate ureteral identification and protect against inadvertent injury in high-risk cases by improving tactile and visual localization. Additionally, meticulous dissection along the left common iliac artery, where the ureter is most closely apposed, along with careful handling of surrounding tissues and judicious use of energy devices, is essential to minimize ischemic and traction-related injury during graft exposure and anastomosis.

More recent radiology literature continues to emphasize the value of delayed-phase imaging as a ‘problem-solving’ step when single-phase CT identifies retroperitoneal fluid, hydronephrosis, delayed nephrogram, or periureteral stranding, as urinary extravasation may be occult on arterial or portal venous phase imaging alone. In these scenarios, CT urography with delayed imaging improves diagnostic confidence by demonstrating opacified urine tracking into a collection, helping differentiate urinoma from hematoma or abscess, and enabling earlier definitive management [10].

Management of urinoma in the setting of a prosthetic vascular graft presents unique challenges. The primary therapeutic goal is urinary diversion away from the site of injury to promote ureteral healing and prevent further leakage. This is commonly achieved through placement of a percutaneous nephrostomy tube, ureteral stent, or both, depending on the severity and location of the injury [10]. In cases where the urinoma lies adjacent to a vascular graft, direct percutaneous drainage is generally avoided because even minimal bacterial contamination can result in graft infection with potentially catastrophic consequences [3,4]. In such scenarios, conservative management with urinary diversion and close imaging surveillance is often preferred [8].

Adjunctive antibiotic therapy is frequently employed when urinomas are located near prosthetic vascular material, even in the absence of documented infection, to minimize the risk of graft contamination [3]. In the present case, multidisciplinary collaboration among urology, vascular surgery, interventional radiology, and infectious disease specialists allowed for a coordinated approach that prioritized graft preservation while effectively treating the ureteral injury. The patient’s favorable outcome underscores the importance of timely diagnosis, appropriate imaging selection, and individualized management strategies.

Earlier recognition of postoperative ureteral injury after aortoiliac reconstruction depends on maintaining suspicion when symptoms are atypical for vascular complications or when imaging shows retroperitoneal collections without clear vascular communication. Clinical triggers include new or progressive flank, lower quadrant, groin, or testicular pain; unexplained hydronephrosis; rising creatinine; persistent ileus; fever or leukocytosis when secondary infection develops; and enlarging retroperitoneal collections on interval studies. When any of these features are present, prompt evaluation with CT urography, including delayed/excretory phase images, or retrograde pyelography when CT is equivocal, can confirm urinary extravasation and accelerate urinary diversion or stenting to limit morbidity [11].

This case highlights two key clinical principles. First, delayed-phase CT urography should be considered when evaluating retroperitoneal fluid collections following vascular surgery, particularly when initial imaging findings are inconclusive. Second, early recognition and coordinated multidisciplinary management are essential to preserve renal function and prevent graft-related complications in patients with suspected ureteral injury after vascular bypass procedures.

## Conclusions

Urinoma formation following aortobifemoral bypass is an uncommon but clinically significant complication that requires a high index of suspicion and appropriate imaging for timely diagnosis. In this case, sequential CT angiography followed by delayed-phase CT urography enabled accurate identification of a ureteral injury and guided effective management. Early urinary diversion with nephrostomy and subsequent ureteral stenting, combined with targeted antibiotic therapy, resulted in complete resolution of the urinoma while preserving renal function and protecting graft integrity. Successful outcomes in such cases depend on close multidisciplinary collaboration among urology, vascular surgery, interventional radiology, and infectious disease teams. This case highlights the importance of delayed-phase CT urography in evaluating postoperative retroperitoneal fluid collections and supports prioritizing urinary diversion over direct drainage for collections adjacent to vascular grafts in the absence of infection or progression.
